# Sarcoptic mange in wild quichua porcupines (*Coendou quichua* Thomas, 1899) in Colombia

**DOI:** 10.1016/j.ijppaw.2018.02.002

**Published:** 2018-02-15

**Authors:** Viviana Gonzalez-Astudillo, Omar D. Leon-Alvarado, Paula Andrea Ossa-Lopez, Fredy Arvey Rivera-Paez, Héctor E. Ramírez-Chaves

**Affiliations:** a School of Veterinary Science, Building 8114, The University of Queensland, Gatton Campus, Queensland, Australia, 4343; b Laboratorio de Sistemática y Biogeografía, Escuela de Biología, Facultad de Ciencias, Universidad Industrial de Santander, Cra. 27 Calle 9 A.A. 678, Bucaramanga, Colombia; c Departamento de Ciencias Biológicas, Universidad de Caldas, Calle 65 # 26-10, Manizales, Colombia

**Keywords:** Alopecia, Andes, Colombia, Rodentia, *Sarcoptes scabiei*, PCR

## Abstract

The Quichua porcupine (*Coendou quichua*) is a neotropical rodent with uncertain taxonomic and conservation status. Two Quichua porcupines with severe hyperkeratosis and alopecia were found in the Magdalena River Basin of Colombia. *Sarcoptes scabiei*, the mite causing mange, a disease carried mainly by domestic animals, was confirmed via parasitological and molecular methods. This is the first report of mange in neotropical porcupines to date. The population-level impact of mange in *Coendou* spp., related mammals and predators in Colombia might represent a threat and needs further investigation.

## Highlights

• Pathological characterisation of sarcoptic mange in cryptic *Coendou quichua* is carried out.
• The impact of sarcoptic mange on *Coendou quichua* is currently unknown.
• New data on *Sarcoptes* hosts carries significance for the economy and conservation.
• Sarcoptic mange is of low priority in Colombia, affecting the understanding of the disease.

## Graphical abstract

**Figure:**
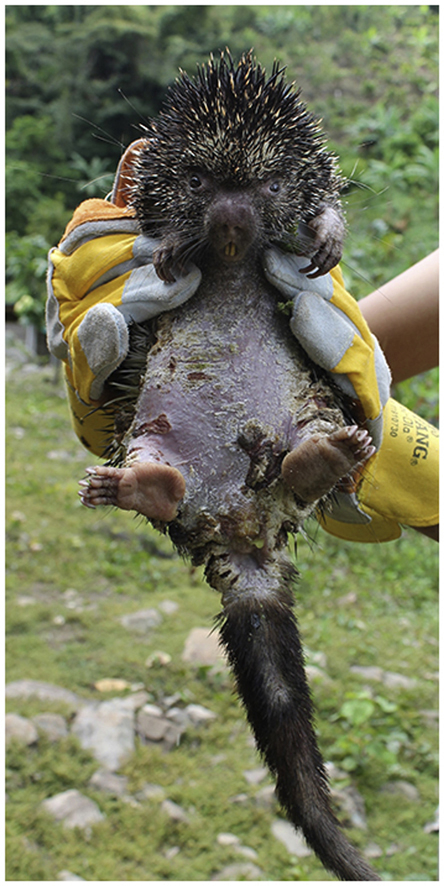

Mites from the genus *Sarcoptes* (species *scabiei*) cause sarcoptic mange, a highly-contagious disease detected in various hosts ranging from humans, to domestic animals and wildlife (Bornstein et al., 2001). Mange can cause negative economic impacts (Bornstein et al., 2001), particularly in countries lacking effective surveillance systems leading to underreporting of animal cases (Cediel et al., 2013). *Sarcoptes scabiei* appears to be one highly diverse species according to microsatellite analyses (Arlian and Morgan, 2017). Consequently, it is thought that cases of wildlife mange are the result of an infection by a single highly variable species displaying physiological specificity across many hosts (Pence et al., 1975). The main morphologic features of *S. scabiei* include tooth-like denticles and club-like setae on the mid-dorsal and posterior idiosoma, respectively (Pence and Ueckermann, 2002). Currently, molecular markers can be used to detect and genetically evaluate the presence of *S. scabiei* in domestic animals, wildlife and humans (Alasaad et al., 2014; Angelone-Alasaad et al., 2015). Clinical manifestations are dictated by the type and magnitude of innate, humoral and cellular responses to mite proteins (Arlian and Morgan, 2017). Two main forms of the disease are recognized depending on the host response, ordinary (protective) and severe crusted mange (pathological). Pathogen 'spillover' to naive hosts can induce marked pathological host responses manifesting in the severe crusted mange form.

Mange is rated as a low priority, emerging zoonosis in Colombia (Rentería, 2008). Because of this, compilation of data regarding prevalence of sarcoptic mange or any other mite infection in the country in human and domestic animals is challenging. Domestic animal cases are not required to be reported to veterinary authorities, leading to scant availability of official animal health data. Only a few wildlife cases are publicly available, the most recent on a cervid (*Mazama* sp.; ICA., 2012). However, parasitological surveys in Colombia have produced a plethora of mite species records, compiled in a recent systematic literature review (Gonzalez-Astudillo et al., 2016), but primarily restricted to other vertebrate species. From the 11 mammalian taxonomic orders analysed in the report (data not shown), 31 mite species were documented in chiropterans - the most extensively sampled order, and one in primates. In contrast, rodents - the second most extensively sampled order, reported no mite records.

*Coendou quichua* is a medium-sized porcupine, distributed in western Colombia, Panama and Ecuador, from sea level to 3300 m (Voss, 2015, Ramírez-Chaves et al., 2016). *C. quichua* is classified as Data Deficient according to the International Union for the Conservation of Nature (Delgado, 2016). Although little is known about its natural history in Colombia, *C. quichua* is mostly arboreal but may use natural burrows, is solitary and nocturnal, with a diet mainly of leaves and fruits (Voss, 2015).

A mature male Quichua porcupine was live-trapped in 09/2014 in San Vicente de Chucurí, Eastern Cordillera range (N 6°47′33.27″, W 73°28′48.23″, 1250m), an area with mixed crops and forest patches. The porcupine was humanely euthanized in the field due to an extremely poor prognosis. The second case involved a mature female trapped in the city of Bucaramanga (N 7°08′, W 73°00′, 959 m) which died under veterinary care and was submitted to the local School of Biology on 06/2016.

In both specimens, gross lesions corresponded to hyperkeratosis, and extended ventrally from the cervical region and caudally towards the proximal portion of the tail and above the tarsal and carpal regions; the skin presented regionally extensive alopecia with broken quills. The alopecic area appeared thickened, wrinkled, and with multifocal white to straw-coloured encrustations. The skin covering articulated regions presented cracks exposing the dermis (Fig. 1a–b). Skin sections and scrapings were submitted to the Universidad Nacional de Colombia (Bogotá) and to Universidad del Valle (Cali) for histopathological assessment and parasitological identification. In the first porcupine, severe changes resembled a moderately lymphoplasmacytic dermatitis with severe hyperkeratosis and pustules. The epidermis was moderately hyperplastic with orthokeratotic and parakeratotic hyperkeratosis. Epidermal crusting with intralesional bacterial erosion and sloughing with intra- and subcorneal epidermal pustules were also observed. Dermal lesions were mild and corresponded to hydropic change, spongiosis, lymphoplasmacytic and mast cell infiltrate and lymphatic dilation. The histopathological findings of the second porcupine paralleled the description of the first individual, but were more severe, and included a marked epidermal necrosis and epidermolysis with embedded quill and keratin fragments. In both porcupines, evidence of multiple intralesional mite structures were found, characterised by a cuticle with peripheral and dorsal horns, hatched egg fragments and intraepidermal coalescing tunnels (Fig. 2a–b). Secondary infection by cocci is considered common in severe mange (Verdugo et al., 2016), likely incited by the excessive epidermal sloughing and other disturbances caused by the mite invasion which can also debilitate the host. Mites were identified as *S. scabiei* using amplification and sequencing of fragments of mitochondrial 16S rDNA, using universal primers for the amplification of *S. scabiei* with an estimated size of 135 bp (Angelone-Alasaad et al., 2015). Both samples were individually submitted for DNA extraction, using the DNeasy Blood and Tissue kit (Qiagen), following the manufacturer's protocol. Extracted DNA samples were tested by PCR, using the forward primer SSUDF (5′-GGGTCTTTTTGTCTTGGAATAAA-3′) and reverse primer SSUDR (5′-CTAAGGTAGCGAAATCATTAGC-3′). PCR products were purified with the QIAquick PCR purification kit (Qiagen) and sent to the Universidad de Los Andes (Bogotá, Colombia) for DNA sequencing. GenBank nucleotide sequence accession numbers for the partial sequences generated in the present study are **MG645006** (San Vicente) and **MG645007** (Bucaramanga). The sequences were analyzed using Basic Local Alignment Search Tool (BLAST; Altschul et al., 1990) to determine the closest similarities with other mite species.

**Fig. 1:**
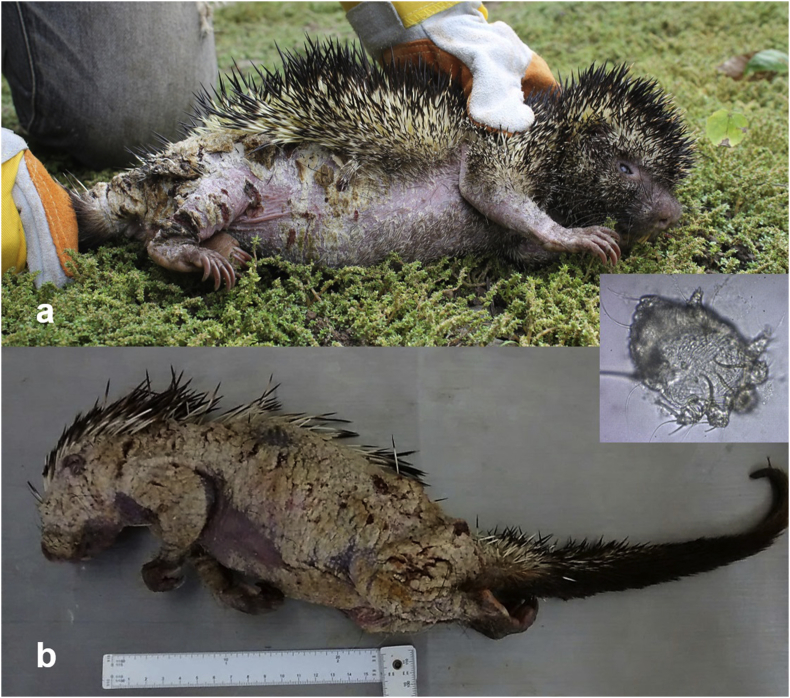
a-b. Macroscopic lesions of severe sarcoptic mange in two Quichua porcupines (*Coendou quichua*) from central Colombia with a microphotograph of *Sarcoptes scabiei* (inset). a) Mature male with extensive alopecia affecting ventral, inguinal region, and limbs. b) Mature female with severe hyperkeratosis and alopecia covering 80% of the body.

**Fig. 2:**
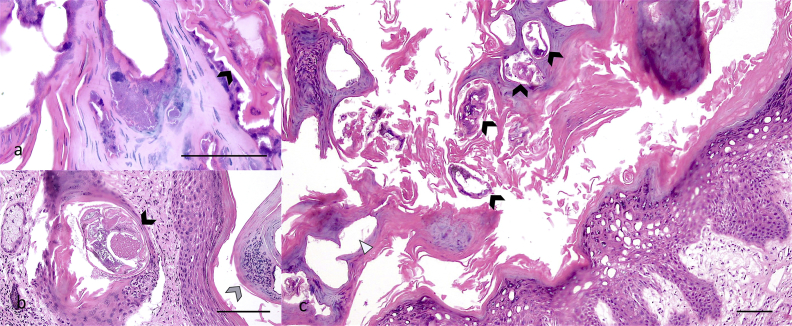
a-c. Microphotograph of skin section of a mature female *Coendou quichua* with severe sarcoptic mange. a) Intralesional cocci observed surrounding parasitic tunnels, with mite (arrow head 20X). b) Intralesional mite (black arrow head) and pustule (grey arrow head 10X), and c) several tunnels with (black arrow heads) and without (white triangle) mites in the stratum corneum (4X). All bars = 100 μm. Haematoxylin and Eosin stain.

The partial sequences of 16S were 100% and 98% (San Vicente de Chucurí and Bucaramanga, respectively) identical to the corresponding sequences available for *S. scabiei* in GenBank isolated from the domestic dog (*Canis lupus familiaris*; KJ781373.1), the Argentine Gray fox (*Lycalopex griseus*; KT223563.1), the European rabbit (*Oryctolagus cuniculus*; AB779577.1), and the buffalo (*Bubalus bubalis*; AB779569.1). Of these, *L. griseus* is not present in Colombia and it is restricted to Patagonia (Hunter, 2011) whereas the others are domestic and/or feral species in the country (Ramírez-Chaves et al., 2011). In particular, domestic dogs are distributed throughout Colombia (Baptiste et al., 2010), where they overlap with *C. quichua* habitat. Dogs have high urban and rural densities (Hughes-David and Macdonald, 2013), and are currently recognized nationally as wildlife predators, disease carriers and competitors (Manjarres-Rodriguez, 2013). Despite the wide host range of *Sarcoptes*, there are no records of mange in neotropical porcupines, likely due to underreporting (Gonzalez-Astudillo et al., 2016, Verdugo et al., 2016). Other arachnids have been reported in Colombian porcupines; specifically, *Amblyomma longirostre* in *Coendou* spp. and *C. prehensilis* (Osorno-Mesa, 1940, Wells et al., 1981), *A. longirostre*, and *A. goaeji* in *Coendou* spp. (CIAT, 1973). The only records available in the literature of mange in porcupines are from a nearctic species, namely *Erethizon dorsatum*, by *S. scabiei* (Payne and O'Meara, 1958) and *Notoedres douglasi* (Snyder et al., 1991). Lesions in these reports did not have such an extensive alopecia, and were mostly ventral-inguinal and facial. Chronic mange induces intense pruritus and alopecia, demanding energy for thermoregulation and activating an immune response, eventually causing a progressive loss of condition (Newman et al., 2002) from high parasitic demands.

Colombian animal health authorities would benefit from developing a wildlife disease surveillance program (Peñuela-Gómez et al., 2012, Gonzalez-Astudillo et al., 2016), establish functional reporting networks and promote research that assesses the impact of domestic animal diseases in wildlife. Considering the epizootiology of the disease and the distribution of the host, the authors suggest the likely cross-species transmission from the domestic dog due to overlapping habitats. Demodectic mange by *Demodex* spp. (demodicosis) is the most prevalent dog mite infection in Colombia and in the metropolitan area where these porcupines were captured (unpublished data) with only sporadic demodectic ‘spillovers’ to the crab-eating fox *Cerdocyon thous* (pers. comm. Dr. J.F. Chica-Galeano). It is suggested that the unknown immunological status of the porcupines, low receptivity to *Demodex* spp. (Singh and Dimri, 2014), wide range of hosts infected with *S. scabiei,* or a possible environmental contamination of burrows with *S. scabiei* shared asynchronously with infected hosts (Skerratt, 1998), contributed to the infection and manifestation of sarcoptic over demodectic mange in these cases. The mange cases presented here could be the result of a few endemic events in a species not much is known of, represent a recent ‘spillover’ from a domestic host, or an ongoing epizootic. During a mange epizootic in naïve populations, the number of cases appear to be devastating (Leon-Vizcaino et al., 1999, Martin et al., 2018); however, most wildlife populations will recover in the long-term. Although mange is currently considered as one of the ten most devastating wildlife diseases (Tompkins et al., 2015), only in a few instances, have entire populations become extinct (Pence and Ueckermann, 2002). To the best of our knowledge, these are the first reports of mange in any neotropical porcupine species. The limited information available for *C. quichua* and of mite infections in Colombia hinders further discussion of the plausible impacts of sarcoptic mange in the long-term conservation of the species.

## Declaration of interest

None.

## Appendix A Supplementary data

Data Profile Data Profile
